# Developmental Changes in the Effect of Active Left and Right Head Rotation on Random Number Generation

**DOI:** 10.3389/fpsyg.2018.00236

**Published:** 2018-02-28

**Authors:** Charlotte Sosson, Carrie Georges, Mathieu Guillaume, Anne-Marie Schuller, Christine Schiltz

**Affiliations:** Institute of Cognitive Science and Assessment, Research Unit Education, Culture, Cognition and Society, Faculty of Language and Literature, Humanities, Arts and Education, University of Luxembourg, Esch-sur-Alzette, Luxembourg

**Keywords:** numerical cognition, embodied cognition, random number generation, active head rotation, developmental changes, children

## Abstract

Numbers are thought to be spatially organized along a left-to-right horizontal axis with small/large numbers on its left/right respectively. Behavioral evidence for this mental number line (MNL) comes from studies showing that the reallocation of spatial attention by active left/right head rotation facilitated the generation of small/large numbers respectively. While spatial biases in random number generation (RNG) during active movement are well established in adults, comparable evidence in children is lacking and it remains unclear whether and how children’s access to the MNL is affected by active head rotation. To get a better understanding of the development of embodied number processing, we investigated the effect of active head rotation on the mean of generated numbers as well as the mean difference between each number and its immediately preceding response (the first order difference; FOD) not only in adults (*n* = 24), but also in 7- to 11-year-old elementary school children (*n* = 70). Since the sign and absolute value of FODs carry distinct information regarding spatial attention shifts along the MNL, namely their direction (left/right) and size (narrow/wide) respectively, we additionally assessed the influence of rotation on the total of negative and positive FODs regardless of their numerical values as well as on their absolute values. In line with previous studies, adults produced on average smaller numbers and generated smaller mean FODs during left than right rotation. More concretely, they produced more negative/positive FODs during left/right rotation respectively and the size of negative FODs was larger (in terms of absolute value) during left than right rotation. Importantly, as opposed to adults, no significant differences in RNG between left and right head rotations were observed in children. Potential explanations for such age-related changes in the effect of active head rotation on RNG are discussed. Altogether, the present study confirms that numerical processing is spatially grounded in adults and suggests that its embodied aspect undergoes significant developmental changes.

## Introduction

Knowledge and thinking are constrained by sensory-motor processes in that motor activities and other sensory-bodily experiences influence the cognitive processing of abstract concepts (Barsalou, 2008). The idea of such “embodied cognition” has become increasingly influential and numerical thinking can be considered as one principle example of it (Lakoff and Nunez, 2000).

According to the hierarchical model by Fischer and Brugger (2011; see also, Fischer, 2012), number processing is characterized by grounded, embodied and situated aspects. *Grounded numerical cognition* refers to the idea that numerical representations reflect the universal laws of the physical world in that small/large numbers are associated with lower/upper space respectively. This is supported by the observation that priming words linked to the lower (e.g., submarine) and upper (e.g., eagle) vertical space with small and large numbers respectively facilitated their treatment (Lachmair et al., 2014). *Embodied numerical cognition* is built on the basis of grounded cognition and suggests that number knowledge depends on spatial-directional learning experiences constituted by specific motor activities and other bodily sensory experiences. One example is the influence of finger counting habits on the association between numerical and spatial representations in healthy adults. While individuals who started counting on their left hand reliably associated small/large numbers with the left/right space respectively, no such effect was observed in right-starters (Fischer, 2008). Finally, *situated numerical cognition* suggests that number-space associations can be directly modulated by the current constraints and context of a situation, including both external stimuli as well as body posture. This level of knowledge representation is very flexible and instantly adapts to concurrent task demands. In that vein, Eerland et al. (2011) reported that participants’ numerical estimates were slightly smaller/larger when they were leaned toward the left/right respectively. Moreover, Loetscher et al. (2008) reported an effect of active motion on random number generation (RNG). Namely, participants produced more smaller/larger numbers while rotating their head toward the left/right respectively (see also Winter and Matlock, 2013).

The effect of movement on numerical production can be explained by spatial attention shifts along a hypothetical mental number line (MNL). According to the MNL hypothesis (for reviews see Dehaene, 1997; Hubbard et al., 2005), numbers are spatially represented along a horizontal axis with small/large numbers on its left/right respectively. The idea of the MNL was initially proposed following the observation of the *spatial–numerical association of response codes (SNARC) effect*, describing faster left-/right-sided responses for small/large digits respectively in binary classification tasks (Dehaene et al., 1993; Hoffmann et al., 2014; Georges et al., 2016). Motion-induced spatial attention shift on this so-called MNL would then bias the access to numerical magnitude representations, thereby explaining the effect of active head rotation on number selection (see Fischer and Shaki, 2014).

The robustness of the effect of motion on the reallocation of attention along the MNL was also further confirmed using bodily effectors other than the participant’s head. For instance, Loetscher et al. (2010) reported that the generation of smaller/larger numbers was preceded by left-/rightward eye movements respectively. Moreover, the selection of numbers during RNG depended on the direction of passive whole-body motion (Hartmann et al., 2012). In addition, Shaki and Fischer (2014) indicated that participants generated more small/large numbers when actively preparing to turn left-/rightward respectively. Interestingly, individuals were also more likely to turn to the left/right following the generation of a small/large number respectively. Cheng et al. (2015) also found that left-lateral arm turns facilitated the generation of smaller numbers relative to right-lateral turns. These findings thus collectively highlight the influence of motion on number processing in healthy adults, thereby providing evidence for the close link between numerical and spatial representations and the situatedness of their associations.

Nonetheless, despite the substantiation of situated numerical cognition in healthy adults, equally compelling evidence in children is sparse. To our knowledge, only Göbel et al. (2015) investigated the effect of spatially directional cues on RNG in 5- to 11-year old children. Concretely, they observed that lying on the left/right side of the body increased the generation of smaller/larger numbers respectively. It thus seems that directional cues can influence numerical production also in children, similarly to adults. However, it remains to be determined whether the generation of numbers at such earlier developmental stages can also be biased by active head rotation, as it has been repeatedly observed in healthy adults (Loetscher et al., 2008; Winter and Matlock, 2013; Cheng et al., 2015). Addressing this question should advance our understanding of spatial-numerical mappings in elementary school children and inform us on how their situatedness develops over the lifespan.

### Aims

In the present study, we therefore aimed to determine the effect of active left/right head rotation on RNG not only in adults, but also in children. Children were recruited from 2nd, 3rd, and 4th grade of elementary school to be in line with the age range of the participants assessed in the study of Göbel et al. (2015), measuring the effect of spatially directional cues on RNG in 5- to 11-year-old children. This should enable us to replicate previous observations in adults and additionally inform us about whether the recently reported effect of static body position on RNG in children (Göbel et al., 2015) can be extended to active head rotation.

Finding evidence for an effect of active left/right head rotation on RNG not only in adults, but also in 7- to 11-year-old elementary school children would highlight potential similarities in spatial-numerical representations as well as in their situatedness across both age groups. In addition, it would suggest that the recently reported spatial bias in RNG observed in younger individuals (Göbel et al., 2015) is not specifically related to static body position. Conversely, the absence of an effect of active left/right head rotation on number processing in children but not adults might indicate developmental changes in the spatial representation of numerical magnitudes. This would then be in line with studies indicating that estimation patterns on the number line task were fitted best by a logarithmic and linear function in children and adults respectively, suggesting an age-related log-to-linear shift in the representation of numerical magnitudes on the MNL (Booth and Siegler, 2006; Moeller et al., 2009). Alternatively, a potential null effect in children might suggest that these younger individuals do not yet activate spatial-numerical associations in tasks such as RNG, which do not involve any explicit magnitude judgments (e.g., van Galen and Reitsma, 2008). Furthermore, age-related differences in the effect of active head rotation on RNG could highlight potential developmental changes in the accessibility of the MNL. Children as opposed to adults might for instance not yet anchor number-space mappings onto an external reference frame when randomly generating numbers during head rotation (Crollen and Noël, 2015; Crollen et al., 2015; Nava et al., 2017). Finally, a potential null effect in the younger individuals could also simply be explained by the current paradigm of randomly generating numbers while actively rotating one’s head. This dual-task scenario might compromise the working memory (WM) resources necessary for MNL activation, especially in children whose executive functions have not yet fully developed (Luciana and Nelson, 1998; De Luca et al., 2003; Best et al., 2009). This could then explain potential differences between the present outcomes and the previous findings by Göbel et al. (2015), who observed an effect of static body position on RNG already in elementary school children.

To quantify the effect of active head rotation on RNG, we computed (a) the mean of generated numbers and (b) the mean difference between each randomly generated number and its immediately preceding response, i.e., the first order difference (FOD). While the mean of generated numbers yields information about overall numerical selection preferences, the mean of FODs provides valuable insights into the way in which the generated numbers are selected on the MNL. More concretely, negative/positive FODs (reflecting descending/ascending steps in the generated numerical sequence) are indicative of left-/rightward spatial attention shifts along the MNL respectively, while smaller/larger FODs in terms of absolute value reflect narrow/wide spatial attention shifts along the MNL respectively regardless of direction.

The means of generated numbers and FODs are commonly used when studying spatial biases in RNG (e.g., Hartmann et al., 2012; Thompson et al., 2013; Winter and Matlock, 2013; Shaki and Fischer, 2014; Göbel et al., 2015). However, the sign (positive/negative) and absolute value (small/large) of FODs carry distinct information regarding spatial attention shifts along the MNL, namely their direction (left-/rightward) and size (narrow/wide) respectively. The relative contribution of these two factors to the overall mean of FODs consequently needs to be disentangled. Concretely, assessing whether e.g., a negative mean of FODs reflects (a) the generation of a higher total of descending steps (i.e., negative FODs) than ascending steps (i.e., positive FODs) and/or (b) the production of larger descending steps than ascending steps in terms of absolute value allows us to investigate more thoroughly how active head rotation affects the reallocation of spatial attention along the MNL. In addition to reporting overall FOD values, we therefore assessed the effect of active left/right head rotation on the total of negative and positive FODs regardless of their numerical values as well as on the absolute value of negative and positive FODs.

## Materials and Methods

The experiment was reviewed and approved by the Ethics Review Panel of the University of Luxembourg. Adults signed a consent form and parental consent was obtained for the children prior to the start of the study.

### Participants

#### Children

In total, seventy children (36 female; mean age = 9.45 years; *SD* = 1.10; range = 7.8–11.9) were recruited from three Luxemburgish public elementary schools from the second (*N* = 21; age = 8.11; *SD* = 0.35), third (*N* = 18; age = 9.38; *SD* = 0.60) and fourth grade (*N* = 31; age = 10.4; *SD* = 0.61). None of the children had a history of learning disorders, such as dyslexia or dyscalculia. Data from the children reported in the present study were part of data collected in the framework of a bigger project including additional tasks not described hereafter.

#### Adults

Twenty-four participants (19 female; age = 23.3 years; *SD* = 4.2; range = 18–34) were recruited at the University of Luxembourg. They received a small compensation in exchange for their participation. None of them had a history of learning disorders, such as dyslexia or dyscalculia. They were all blind to the hypotheses of the experiment.

### Procedure

Participants were asked to orally generate numbers between 1 and 30 as randomly as possible. To assist the participants in their understanding of “as randomly as possible,” we added the following sentence: “Imagine you have a bag in which there are thirty balls numbered 1–30 and whenever instructed you have to take a ball from the bag and tell me which number you see. After having said the number, you have to return the ball to the bag.” Subjects had to do this task while moving their head from left-to-right (i.e., right rotation) and from right-to-left (i.e., left rotation). They had their eyes covered with a mask during the entire task to prevent any distractions from their surroundings. The starting position of the head (head above left vs. right shoulder) was counterbalanced across participants. Participants were asked to generate the number halfway through their motion (i.e., when their head was aligned with their trunk), as opposed to when their head was fully turned toward the left/right side and as such had reached a static position (as in e.g., Loetscher et al., 2008). This was to clearly differentiate the current paradigm, investigating the effect of active head motion on RNG, from that of Göbel et al. (2015), who studied the effect of static left/right body position on numerical processing in children. The starting of the head movement was announced through a beep given via a headset every 3.6 s. The average speed of the motion was therefore 0.14 Hz (i.e., one turn per 7.2 s). Rotational speed was slowed down compared to Loetscher et al. (2008) to provide the participants with sufficient time to generate numbers during active head rotation and to minimize the total of omissions and errors especially in the younger participants. The script of the generation task was running on Matlab on an 11-in. MacBook and the responses were recorded on a Maxxter Stereo Headset.

As previously done by Loetscher et al. (2008), 40 numbers had to be generated per condition (left, right), which resulted in 80 numbers in total. The session was divided into two blocks, thus resulting in 40 trials per block. To ensure that participants understood the task, a training session consisting of 16 trials preceded the actual experiment.

### Data Analysis

First, we analyzed the total of ***omissions*** and ***errors*** during RNG. Responses were considered as erroneous if the generated number was outside the 1–30 range. We also quantified the overall randomness of number generation by computing the ***redundancy score*** (R score; Evans, 1978). The R score reflects the extent to which each response is generated with equal frequency. A score of 0% implies no redundancy, while a score of 100% indicates complete redundancy (i.e., all responses are identical). The latter calculation was achieved by a published computer program, freely downloadable at http://www.lancs.ac.uk/staff/towse/rgcpage.html (Towse and Neil, 1998). Assessing the effect of age group (adults vs. children) on these measures should inform us about potential age-related differences in overall task comprehension and performance. We also determined whether the total of omissions and errors during RNG depended on active left/right head rotation and/or its interaction with age group. It should be noted that left/right rotation refers to the left-/rightward motion during which the selected number had to be produced. Conversely, since measures of randomness in RNG, such as the R score, are not believed to rely on or directly index any numerical magnitude representations (Brugger, 1997), but supposedly predominantly depend on more general executive functions (Brugger, 1997; Baddeley, 1998; Peters et al., 2007; Terhune and Brugger, 2011), we did not assess the effect of rotation on the redundancy score.

To measure the effect of active head rotation on RNG, we referred to the study of Winter and Matlock (2013) and analyzed all correctly generated numbers as a continuous measure rather than binning them according to their magnitudes (i.e., smaller or larger than the mean of the number range; as in Loetscher et al., 2008). Two analyses were conducted based on this measure.

In a first step, we determined whether ***the mean of correctly generated numbers*** in each participant differed between active left/right head rotation.

In a second step, we focused on the arithmetic difference between each generated number and its immediately preceding response (i.e., the first order difference; FOD) and determined whether ***the mean of FODs*** in each participant differed between active left/right head rotation. In case a response was omitted or outside the 1–30 range, the FODs between this incorrect/omitted response and its preceding as well as succeeding number were discarded from data analyses.

In general, FODs can be classified depending on two factors: (1) their sign (positive vs. negative) and (2) their absolute numerical value (small vs. large). In spatial terms, the sign of the FOD reflects the direction of the step on the MNL. While positive FODs, indexing an ascending step in the generated numerical sequence, correspond to a “rightward” shift along the MNL, negative FODs, reflecting a descending step in the generated numerical sequence, correspond to a “leftward” shift on the MNL. Conversely, the absolute numerical value of the FOD reflects the size of the step on the MNL regardless of its ascending or descending direction. The overall mean of FODs thus depends on the interplay between these two factors.

To disentangle the relative contribution of these two factors to the mean of FODs, we performed two additional analyses.

Firstly, we determined whether *the total of FODs* differed depending on their positive/negative sign – henceforth referred to as “direction” as it reflects the ascending/descending direction of the step in the generated numerical sequence – during left/right rotation. In other terms, we assessed the effects of direction and rotation on the total of FODs. More concretely, we compared the total of positive and negative FODs during both left and right rotations regardless of their numerical value. We hypothesized that positive FODs should outnumber negative FODs during right rotation, but vice-versa during left rotation. Moreover, participants should generate more positive/negative FODs during right/left than left/right rotations respectively. It should be noted that the total of FODs is a continuous variable ranging from 0 to 39 during both left and right rotations (i.e., the total of 40 numbers generated per left/right rotation minus one). In addition, it is worth mentioning that the totals of positive and negative FODs should in theory be inversely proportional. More concretely, more positive FODs should be associated with less negative FODs such that the total of FODs always adds up to 39. Nonetheless, this was practically not the case in the present investigation considering the exclusion of FODs preceding as well as succeeding erroneous and omitted responses. Moreover, FODs of zero, resulting from the repetition of the same number on two (or more) consecutive trials, could not be considered for the current analysis. The totals of positive and negative FODs thus ranged from 24 to 39 and 27 to 39 during left and right rotation respectively, entailing that negative and positive FODs were practically not directly inversely proportional in the present study. As such, it is important to include “direction” as an additional factor in the ANOVA rather than simply assessing only the effect of rotation on either positive or negative FODs.

Secondly, we ascertained whether direction and/or rotation affected *the mean absolute value of FODs*. More concretely, we compared the means of positive and negative FODs in terms of absolute value during both left and right rotations. We hypothesized that positive FODs should be larger in terms of absolute value than negative FODs during right rotation, but vice-versa during left rotation. Moreover, participants should perform larger positive/negative FODs (in terms of absolute value) during right/left than left/right rotations.

To measure potential age-related changes in the effect of active head rotation on RNG, the above analyses were conducted including age group (adults vs. children) as between-subject factor. In case of a significant interaction between age group and one of the within-subject variables (i.e., rotation and direction), two separate ANOVAs were subsequently performed – one for each age group. When only focusing on the subgroup of children, grade was additionally added as a between-subject factor in all the analyses. This was mainly to exclude the possibility that any potential interaction effects between age group and the within-subject variables rotation and/or direction on the different dependent variables were driven only by a certain grade.

Considering that individuals’ counting strategies might be affected by their initial starting position (see Towse and Cheshire, 2007), each of the following analyses was initially conducted including starting orientation as an additional between-subject variable. Since starting orientation did, however, not have any main or interaction effects, we decided to drop this variable from data analysis. All analyses reported below were thus conducted without starting orientation as between-subject factor.

An alpha of 0.05 was used as the cut-off for significance (i.e., the null hypothesis was rejected if *p* < 0.05) in all the following analyses.

## Results

All descriptives can be found in **Table 1**.

**Table 1:** Descriptive information.

Variables	Children		Adults	
		
	Left rotation	Right rotation	Left rotation	Right rotation
Total of omissions	0.94 (1.52)	0.96 (1.44)	0.04 (0.20)	0.04 (0.20)
Total of errors	0.6 (1.17)	0.8 (1.50)	0	0
*R* score	10.64 (6.40)		7.52 (4.20)	
Mean of generated numbers	14.14 (2.46)	14.08 (2.46)	13.63 (1.92)	14.34 (1.80)
Mean of FODs	0.27 (1.49)	0.23 (1.45)	-0.69 (1.62)	0.93 (1.50)
Total of negative FODs	14.74 (4.61)	14.44 (4.09)	17.71 (4.62)	14.83 (3.84)
Total of positive FODs	21.39 (5.08)	21.84 (4.03)	21.13 (4.47)	24.00 (3.74)
Mean absolute value of negative FODs	7.14 (2.44)	8.13 (3.03)	9.82 (3.02)	8.59 (2.96)
Mean absolute value of positive FODs	5.53 (1.94)	5.66 (2.11)	6.70 (2.13)	6.98 (1.81)


*Standard deviations are shown in parentheses. The *R* score was only compared between the different age groups, but not between left-/right-sided rotation.*

### Preliminary Analyses

#### The Total of Omissions as a Function of Rotation in Children and Adults

A 2 × 2 mixed ANOVA on the total of omissions including rotation and age group as within- and between-subject factors respectively indicated a main effect of age group [*F*(1,92) = 9.97; *p* = 0.002; $\eta_{p}^{2}$ = 0.1], with children omitting responses on significantly more trials than adults (children: $\overset{¯}{x}$ = 1.9; *SD* = 2.8 vs. adults: $\overset{¯}{x}$ = 0.08; *SD* = 0.28). The total of omissions did, however, not differ between left and right rotation [*F*(1,92) = 0.01; *p* = 0.94; $\eta_{p}^{2}$ = 0.00] and there was no interaction between rotation and age group [*F*(1,92) = 0.01; *p* = 0.94; $\eta_{p}^{2}$ = 0.00]. In children, a 2 × 3 mixed ANOVA including rotation and grade as within- and between-subject factors respectively revealed no effect of grade [*F*(2,67) = 0.77; *p* = 0.47; $\eta_{p}^{2}$ = 0.02] and there was no interaction between grade and rotation [*F*(2,67) = 0.78; *p* = 0.46; $\eta_{p}^{2}$ = 0.02].

#### The Total of Errors as a Function of Rotation in Children and Adults

A 2 × 2 mixed ANOVA on the total of errors including rotation and age group as within- and between-subject factors respectively indicated a main effect of age group [*F*(1,92) = 7.48; *p* = 0.01; $\eta_{p}^{2}$ = 0.08], with children generating more numbers outside the 1–30 range than adults (children: $\overset{¯}{x}$ = 1.4; *SD* = 2.5 vs. adults: $\overset{¯}{x}$ = 0.0; *SD* = 0.0). However, there was no main effect of rotation [*F*(1,92) = 0.95; *p* = 0.33; $\eta_{p}^{2}$ = 0.01] and also no interaction between rotation and age group [*F*(1,92) = 0.95; *p* = 0.33; $\eta_{p}^{2}$ = 0.01]. A 2 × 3 mixed ANOVA including rotation and grade as within- and between-subject factors respectively indicated that grade significantly affected the total of errors [*F*(2,67) = 4.00; *p* = 0.02; $\eta_{p}^{2}$ = 0.11]. *Post hoc* pairwise comparisons revealed that 2nd graders generated more numbers outside the specified range than 4th graders [2nd grade: $\overset{¯}{x}$ = 2.43 vs. 4th grade: $\overset{¯}{x}$ = 0.55; *t*(24.46) = 2.57; *p* = 0.02; Cohen’s *d* = 0.77]. There was, however, no interaction between grade and rotation [*F*(2,67) = 0.05; *p* = 0.95; $\eta_{p}^{2}$ = 0.001].

Only correct responses within the 1–30 range (95.9% in children vs. 99.9% in adults) were considered for all subsequent analyses.

#### The Randomness Quality in Children and Adults

The redundancy score across all participants was 9.84 (*SD* = 6.05). A one-way ANOVA including age group as between-subject factor revealed a main effect [*F*(1,92) = 4.94; *p* = 0.03; $\eta_{p}^{2}$ = 0.05], with adults generating more random numerical sequences than children (adults: *R* score = 7.52; *SD* = 4.20 vs. children: *R* score = 10.64; *SD* = 6.40). In children, a one-way ANOVA indicated that the *R* score did, however, not differ depending on grade [*F*(2,67) = 2.75; *p* = 0.07; $\eta_{p}^{2}$ = 0.08].

### The Mean of Generated Numbers as a Function of Rotation in Children and Adults

**FIGURE 1:**
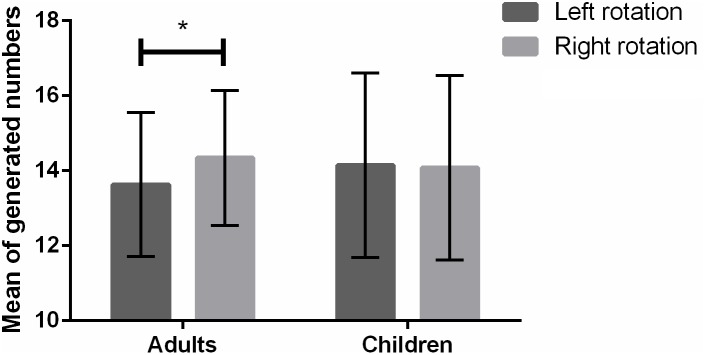
Mean of generated numbers as a function of left/right rotation in adults and children. Error bars represent standard deviation. Asterisks denote statistical significance (^∗^*p* < 0.05).

A 2 × 2 mixed ANOVA on the mean of correctly generated numbers including rotation and age group as within- and between-subject factors respectively did not reveal a main effect of rotation [*F*(1,92) = 3.67; *p* = 0.06; $\eta_{p}^{2}$ = 0.04] or age group [*F*(1,92) = 0.06; *p* = 0.81; $\eta_{p}^{2}$ = 0.001]. Nonetheless, a significant interaction between rotation and age group was observed [*F*(1,92) = 5.08; *p* = 0.03; $\eta_{p}^{2}$ = 0.05]. A follow-up repeated measures ANOVA in adults revealed that the effect of rotation was significant [*F*(1,23) = 5.21; *p* = 0.03; $\eta_{p}^{2}$ = 0.19; see **Figure 1**]. Namely, adults generated on average smaller numbers during left ($\overset{¯}{x}$ = 13.63; *SD* = 1.92) than right rotation ($\overset{¯}{x}$ = 14.34; *SD* = 1.80). Conversely, in children, a follow-up 2 × 3 mixed ANOVA including rotation and grade as within- and between-subject factors respectively did not indicate an effect of rotation [*F*(1,67) = 0.003; *p* = 0.96; $\eta_{p}^{2}$ = 0.00; see **Figure 1**], suggesting that the mean of generated numbers did not significantly differ depending on left ($\overset{¯}{x}$ = 14.14; *SD* = 2.46) or right rotation ($\overset{¯}{x}$ = 14.08; *SD* = 2.46). In the latter participants, there was also no effect of grade [*F*(2,67) = 1.31; *p* = 0.28; $\eta_{p}^{2}$ = 0.04] nor did the interaction between rotation and grade reach significance [*F*(2,67) = 0.76; *p* = 0.47; $\eta_{p}^{2}$ = 0.02].

### The Mean of First Order Differences as a Function of Rotation in Children and Adults

**FIGURE 2:**
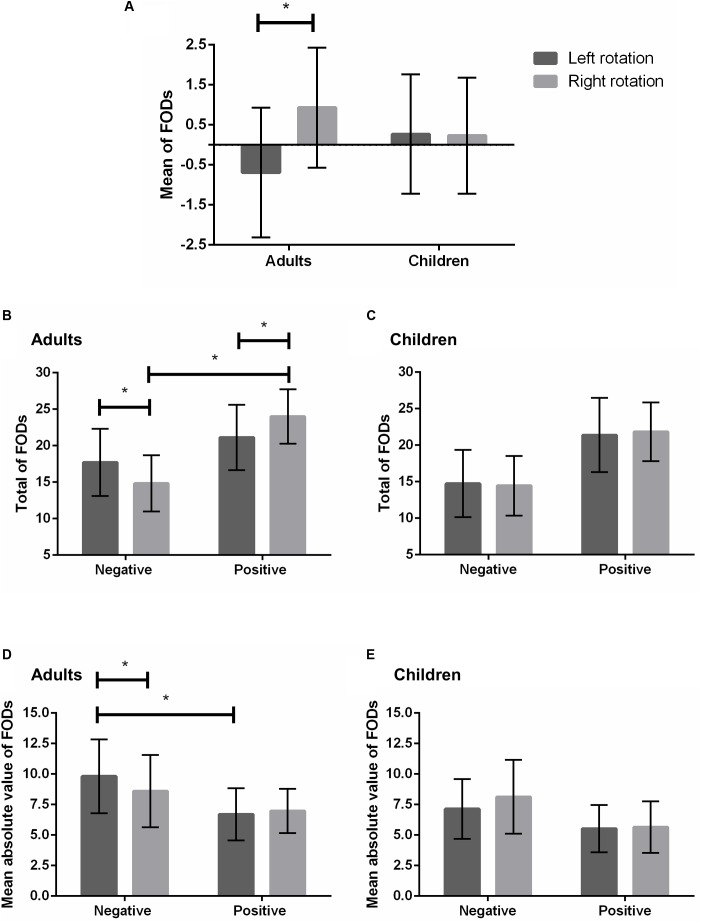
First order differences as a function of left/right rotation in adults and children. The mean of FODs **(A)**. The total of negative and positive FODs in adults **(B)** and children **(C)**. The mean absolute value of negative and positive FODs in adults **(D)** and children **(E)**. Error bars represent standard deviation. Asterisks denote statistical significance (^∗^*p* < 0.05).

A 2 × 2 mixed ANOVA on the mean of the differences between each generated number and its immediately preceding response (i.e., the FOD) including rotation and age group as within- and between-subject factors respectively did not indicate a main effect of age group [*F*(1,92) = 2.67; *p* = 0.11; $\eta_{p}^{2}$ = 0.03]. However, a main effect of rotation [*F*(1,92) = 5.26; *p* = 0.02; $\eta_{p}^{2}$ = 0.05] as well as a significant interaction between rotation and age group [*F*(1,92) = 5.88; *p* = 0.02; $\eta_{p}^{2}$ = 0.06] were revealed. A follow-up repeated measures ANOVA in adults indicated that the effect of rotation was significant [*F*(1,23) = 6.59; *p* = 0.02; $\eta_{p}^{2}$ = 0.22; see **Figure 2A**]. Namely, the mean of FODs was significantly smaller during left ($\overset{¯}{x}$ = -0.69) than right rotation ($\overset{¯}{x}$ = 0.93). Conversely, in children, a follow-up 2 × 3 mixed ANOVA including rotation and grade as within- and between-subject factors respectively revealed no main effect of rotation [*F*(1,67) = 0.008; *p* = 0.93; $\eta_{p}^{2}$ = 0.00; see **Figure 2A**], indicating no significant differences in the mean of FODs depending on left ($\overset{¯}{x}$ = 0.27) or right rotation ($\overset{¯}{x}$ = 0.23). Moreover, there was no main effect of grade [*F*(2,67) = 0.15; *p* = 0.86; $\eta_{p}^{2}$ = 0.01] and no interaction between grade and rotation on the mean of FODs in children [*F*(2,67) = 0.47; *p* = 0.62; $\eta_{p}^{2}$ = 0.01].

#### The Total of First Order Differences as a Function of Direction and Rotation in Children and Adults

A 2 × 2 × 2 mixed ANOVA on the total of FODs including age group as between-subject factor and direction (ascending/positive vs. descending/negative) as well as rotation as within-subject variables did not indicate a main effect of rotation [*F*(1,92) = 0.43; *p* = 0.52; $\eta_{p}^{2}$ = 0.01], but a significant effect of direction was revealed [*F*(1,92) = 66.47; *p* < 0.001; $\eta_{p}^{2}$ = 0.42]. Namely, a higher total of positive than negative FODs was observed across all participants, indicating that individuals generated more ascending than descending steps regardless of age. Moreover, a main effect of age group was revealed [*F*(1,92) = 21.29; *p* < 0.001; $\eta_{p}^{2}$ = 0.19], with the total of FODs being significantly higher in adults than children. This confirms the higher total of omissions and errors in the latter participants (see above).

Most interestingly, however, a significant interaction between direction and rotation was observed [*F*(1,92) = 7.95; *p* = 0.006; $\eta_{p}^{2}$ = 0.08], which additionally depended on age group [*F*(1,92) = 4.68; *p* = 0.033; $\eta_{p}^{2}$ = 0.048]. A follow-up 2 × 2 repeated measures ANOVA in adults including direction and rotation as within-subject factors indicated a significant interaction between these variables [*F*(1,23) = 5.46; *p* = 0.03; $\eta_{p}^{2}$ = 0.19; see **Figure 2B**]. More concretely, the total of negative FODs was higher during left ($\overset{¯}{x}$ = 17.71) than right rotation [$\overset{¯}{x}$ = 14.83; *F*(1,23) = 5.44; *p* = 0.03; $\eta_{p}^{2}$ = 0.19], while positive FODs were more numerous during right ($\overset{¯}{x}$ = 24.00) than left rotation [$\overset{¯}{x}$ = 21.13; *F*(1,23) = 5.47; *p* = 0.03; $\eta_{p}^{2}$ = 0.19]. In addition, positive FODs significantly out-numbered negative FODs during right [positive: $\overset{¯}{x}$ = 24.00; negative: $\overset{¯}{x}$ = 14.83; *F*(1,23) = 35.16; *p* < 0.001; $\eta_{p}^{2}$ = 0.61)] but not left rotation [positive: $\overset{¯}{x}$ = 21.13; negative: $\overset{¯}{x}$ = 17.71; *F*(1,23) = 3.40; *p* = 0.08; $\eta_{p}^{2}$ = 0.13]. As opposed to adults, a follow-up 2 × 2 × 3 mixed ANOVA in children including rotation and direction as within-subject factors and grade as between-subject variable did not indicate an interaction between direction and rotation [*F*(1,67) = 0.69; *p* = 0.41; $\eta_{p}^{2}$ = 0.01; see **Figure 2C**). Positive FODs were more numerous than negative FODs regardless of left [positive: $\overset{¯}{x}$ = 21.39; negative: $\overset{¯}{x}$ = 14.74; *F*(1,67) = 32.09; *p* < 0.001; $\eta_{p}^{2}$ = 0.32] or right rotation [positive: $\overset{¯}{x}$ = 21.84; negative: $\overset{¯}{x}$ = 14.44; *F*(1,67) = 60.23; *p* < 0.001; $\eta_{p}^{2}$ = 0.47]. Moreover, no significant differences between left and right rotations were observed for the totals of negative [*F*(1,67) = 0.45; *p* = 0.50; $\eta_{p}^{2}$ = 0.01] or positive FODs [*F*(1,67) = 0.95; *p* = 0.34; $\eta_{p}^{2}$ = 0.01]. The absence of an interaction between direction and rotation in children did not depend on grade [*F*(2,67) = 0.22; *p* = 0.81; $\eta_{p}^{2}$ = 0.01] and there was no main effect of grade on the total of FODs [*F*(2,67) = 1.55; *p* = 0.22; $\eta_{p}^{2}$ = 0.04].

#### The Mean Absolute Value of First Order Differences as a Function of Direction and Rotation in Children and Adults

A 2 × 2 × 2 mixed ANOVA on the mean absolute value of FODs including age group as between-subject factor and direction as well as rotation as within-subject variables indicated no main effect of rotation [*F*(1,92) = 0.05; *p* = 0.82; $\eta_{p}^{2}$ = 0.001], but a significant effect of direction [*F*(1,92) = 59.87; *p* < 0.001; $\eta_{p}^{2}$ = 0.39]. In general, participants generated larger negative ($\overset{¯}{x}$ = 8.15; *SD* = 2.57) than positive FODs ($\overset{¯}{x}$ = 5.94; *SD* = 1.88) in terms of absolute value (i.e., individuals generally performed larger descending steps). Moreover, a main effect of age group was observed [*F*(1,92) = 11.35; *p* = 0.001; $\eta_{p}^{2}$ = 0.11] in that larger FODs were performed by adults ($\overset{¯}{x}$ = 7.74; *SD* = 1.94) than children ($\overset{¯}{x}$ = 6.22; *SD* = 1.75).

Most importantly, however, we found a significant interaction between direction, rotation and age group [*F*(1,92) = 7.57; *p* = 0.007; $\eta_{p}^{2}$ = 0.08]. A follow-up 2 × 2 repeated measures ANOVAs in adults including direction and rotation as within-subject factors indicated that the interaction between direction and rotation was significant [*F*(1,23) = 4.59; *p* = 0.04; $\eta_{p}^{2}$ = 0.17; see **Figure 2D**]. Namely, the main effect of direction with larger negative than positive FODs was more pronounced during left [negative $\overset{¯}{x}$ = 9.82; positive $\overset{¯}{x}$ = 6.70; *F*(1,23) = 31.65; *p* < .001; $\eta_{p}^{2}$ = 0.58] than right rotation [negative $\overset{¯}{x}$ = 8.59; positive $\overset{¯}{x}$ = 6.98; *F*(1,23) = 7.29; *p* = 0.013; $\eta_{p}^{2}$ = 0.24]. Moreover, a main effect of rotation was observed for negative FODs [*F*(1,23) = 4.66; *p* = 0.04; $\eta_{p}^{2}$ = 0.17] in that the latter were significantly larger in terms of absolute value during left ($\overset{¯}{x}$ = 9.82) than right rotation ($\overset{¯}{x}$ = 8.59). In children, a follow-up 2 × 2 × 3 mixed ANOVA including rotation and direction as within-subject factors and grade as between-subject variable indicated a main effect of rotation [*F*(1,67) = 7.56; *p* = 0.008; $\eta_{p}^{2}$ = 0.10], with children generating larger FODs when turning their heads right- ($\overset{¯}{x}$ = 6.4) compared to leftward ($\overset{¯}{x}$ = 6.04). As opposed to adults, the effect of rotation did, however, not depend on direction [*F*(1,67) = 2.3; *p* = 0.13; $\eta_{p}^{2}$ = 0.03; see **Figure 2E**]. The absence of an interaction between rotation and direction in children was not affected by grade [*F*(2,67) = 1.77; *p* = 0.18; $\eta_{p}^{2}$ = 0.05] and there was no overall effect of grade [*F*(2,67) = 2.56; *p* = 0.08; $\eta_{p}^{2}$ = 0.07].

## Discussion

The present study aimed to determine whether active left/right head rotation biases RNG not only in adults, but also in 7- to 11-year-old elementary school children. This should inform us about whether the recently reported effect of static body position on RNG in children (Göbel et al., 2015) can be extended to active head rotation. Overall, this will further advance our understanding of spatial-numerical mappings in elementary school children and the lifespan development of their situatedness.

In line with previous findings (Loetscher et al., 2008; Winter and Matlock, 2013; Cheng et al., 2015), adults produced on average smaller numbers during left than right rotation. In addition, the mean of FODs was smaller when rotating the head left- as opposed to rightward. Considering that the average FOD potentially depends not only on the total of descending and ascending steps in the generated numerical sequence, but also on their respective absolute values, we additionally studied the effects of rotation on the total as well as the absolute value of negative and positive FODs. This provides further information on how active head rotation affects spatial attention shifts along the MNL. Interestingly, the smaller mean of FODs during left than right rotation reflected the generation of significantly larger descending than ascending steps in terms of absolute value, while the larger mean of FODs during right than left rotation was mainly due to the production of a higher total of ascending than descending steps. When looking at it from a different angle, participants produced more descending steps during left than right rotation, while ascending steps were more numerous when moving the head right- as opposed to leftward. This suggests that participants shifted their attentional focus more often toward the left/right along the MNL when rotating their heads in the left/right direction respectively. In addition, the size of descending steps was larger (in terms of absolute value) during left than right movement. Overall, these findings highlight the close link between numerical and spatial representations, likely encoded in overlapping brain circuits in the posterior parietal cortex and particularly in areas in and around the intraparietal sulcus (for reviews, see Hubbard et al., 2005, 2009; for the “neuronal recycling” hypothesis, see Dehaene, 2005; Dehaene and Cohen, 2007). Moreover, the present findings provide further evidence for the situatedness of spatial-numerical interactions in adults.

Importantly, as opposed to adults, we did not observe a significant influence of active head rotation on RNG in children. The absence of a significant effect in the latter participants did also not depend on grade. Although the absence of evidence for a significant difference in RNG between left and right rotation in children should not be directly considered as evidence of absence of an effect of active head rotation on RNG in the younger participants, the present findings suggest that the spatial bias in RNG during active head motion observed in adults likely only emerges at later developmental stages, at the earliest after 4th grade. In general, the observed null effect in 2nd to 4th graders might have several reasons, which will be discussed in the following paragraphs.

First, the absence of a significant effect of active left/right head rotation on number processing in children might indicate that these younger individuals do not yet represent numerical magnitudes in a spatial format akin to a MNL, as it is likely the case in adults. This assumption is supported by the observation that reading direction affected the orientation of spatial-numerical mappings on the MNL (Shaki et al., 2009), suggesting that number-space associations only gradually arise after formal schooling through reading acquisition (see also, Berch et al., 1999; Zebian, 2005; White et al., 2012). In line with this view, Ninaus et al. (2017) recently observed an age-related increase in the SNARC effect. These findings thus collectively suggest that number-space associations probably only arise later in life through embodied spatially directional experiences such as reading and writing direction.

Nonetheless, the idea that spatial-numerical interactions only arise after formal schooling through reading acquisition was refuted by studies evidencing number-space associations also in preliterate children. Namely, Hoffmann et al. (2013) reported a SNARC effect in a color judgment task already in 5.5-year-old preschoolers. Patro and Haman (2012) even observed a SNARC-like effect in 4-year-old children in that they associated small/large non-symbolic numerosities with the left/right respectively. In addition, most preschoolers already add, subtract and count from left-to-right (Opfer et al., 2010; Opfer and Furlong, 2011; Shaki et al., 2012). Interestingly, left-to-right counting was only observed in children growing up in England, while Palestinian preschoolers mainly counted from right-to-left (Shaki et al., 2012). Number-space associations thus likely emerge much earlier in life through directionally relevant cultural experiences. Interestingly, some studies even reported number-space associations in infants and neonates (de Hevia et al., 2006, 2014a,b; de Hevia and Spelke, 2009, 2010; Lourenco and Longo, 2010), thereby suggesting their innateness. The null effect of active left/right head rotation on number processing in children is thus not likely to be explained by children’s lack of spatial-numerical interactions.

A more likely explanation for the observed discrepancy between adults and children could be developmental changes in the spatial representation of numerical magnitudes. Interestingly, estimation patterns on the number line task were fitted best by a logarithmic and linear function in children and adults respectively, suggesting an age-related log-to-linear shift in the representation of numerical magnitudes on the MNL (Booth and Siegler, 2006; Moeller et al., 2009). Within a logarithmic representation, small numbers are spaced further apart than larger ones (Simms et al., 2016). Children should thus have better access than adults to relatively smaller numerical magnitudes, given their extended representations on the MNL. We did, however, not observe a main effect of age group on the mean of generated numbers, suggesting no age differences in the selection of smaller numerical magnitudes and as such spatial-numerical representations in the current sample. Moreover, previous studies indicated that performances on the 0-to-100 number line estimation task can already be best explained by a linear model from 2nd grade onward (Siegler and Opfer, 2003; Siegler and Booth, 2004). Children in the present study, especially those attending 3rd and 4th grade, thus probably featured mostly linear spatial-numerical representations. Finally, it is also worth noting that although estimation patterns in the number line task are usually interpreted as an indication of the logarithmic or linear nature of numerical magnitude representations (e.g., Siegler and Opfer, 2003; Laski and Siegler, 2007; Opfer and Siegler, 2007), performances on this task might not directly index scaling of the MNL representation in an isomorphic way. Number line estimation performances might more likely index number knowledge (Ebersbach et al., 2008), understanding of the place-value structure (e.g., Moeller et al., 2009), the adoption of certain solution strategies (Barth and Paladino, 2011; Cohen and Blanc-Goldhammer, 2011; Slusser et al., 2013) or attention processes (Anobile et al., 2012). Consequently, rather than reflecting a developmental change in the underlying spatial-numerical representations, the age-related log-to-linear shift in the fit of number line estimation performances might indicate the adoption of different resolution strategies in children and adults. It is therefore unclear whether children and adults feature different spatial-numerical representations. Developmental changes in the latter might thus not be the reason underlying age-related differences in the effect of active head rotation on RNG. Moreover, if this were the case, the effect of head motion on RNG in children should have depended on grade, with 3rd and 4th graders showing similar spatial biases in RNG during rotation than adults, given their already mostly linear numerical magnitude representations.

The null effect in children, as opposed to adults, could, however, potentially be explained by age-related changes in the activation of number-space mappings on the MNL. Children might simply not yet activate spatial-numerical associations in tasks such as RNG, which do not involve any explicit magnitude judgments. This idea is in line with results from van Galen and Reitsma (2008), who observed that younger children only displayed a SNARC effect during explicit magnitude classifications, but not when numerical magnitude information was task-irrelevant during parity judgments. Nonetheless, as already mentioned before, Hoffmann et al. (2013) reported a SNARC effect in a numerical magnitude-irrelevant color judgment task even in preschoolers at the age of 5.5 years. Moreover, Chinese children were shown to display a parity SNARC effect already in Kindergarten at the age of 5.8 years (Yang et al., 2014). The latter findings thus suggest that children activate spatial-numerical representations on the MNL even when numerical magnitude information is not directly task-relevant. As such, inefficient activation of the MNL during RNG in children might not account for the absence of a significant effect of active left/right head rotation on number production in the latter individuals. It should, however, be noted that Göbel et al. (2015) failed to observe a relation between the parity SNARC effect and the spatial bias in RNG in adults, suggesting that these effects might arise from different underlying spatial-numerical representations. As such, evidence for number-space mappings during numerical magnitude-irrelevant parity judgments might not necessarily suggest the activation of spatial-numerical representations also during RNG. Consequently, it cannot be refuted that children, as opposed to adults, did not activate numerical representations on the MNL while randomly selecting numbers in the present study, which could then explain the null effect of active left/right head rotation.

Another possible explanation might be that the activation pattern of spatial-numerical representations does not yet depend on situated factors at earlier developmental stages. Nonetheless, spatially directional cues such as left/right body position were previously shown to increase the generation of smaller/larger numbers respectively already in 5 to 11-year-old children (Göbel et al., 2015). Moreover, simply observing left-to-right or right-to-left reading from storybooks instantaneously affected the counting direction of 3 to 5-year old preliterates in line with the direction of observed reading (Göbel et al., 2017). The activation of spatial-numerical representations on the MNL thus seems to be flexibly modulated by situational demands also in children. The lack of situatedness of spatial-numerical associations in children therefore unlikely explains the current findings.

Children might, however, access their number-space mappings in a different way than adults. Developmental changes in the accessibility of the MNL could then explain age-related differences in the effect of active head rotation on RNG. In this vein, Towse et al. (2014) reported that children featured different number preferences than adults during RNG. Namely, while adults showed a reliable and systematic bias toward the selection of smaller numbers, 8- to 11-year-old children preferentially generated larger numbers. The authors also evidenced a relation between age and the strength of the small number bias, suggesting a developmental increase in the preference for the selection of smaller numerical magnitudes. Adults were also shown to generate both ascending and descending numerical sequences, while children tended to produce mostly ascending sequences (Towse et al., 2014). Reluctance toward the generation of descending steps in children might not only explain their greater preferences for the selection of larger numbers in the study of Towse et al. (2014), but also potentially account for the absence of a significant effect of active head rotation on RNG in the present investigation.

Moreover, children likely anchor number-space mappings onto different spatial reference frames than adults. Namely, 6-year-old children did not display a SNARC effect when their hands were crossed (Nava et al., 2017), while sighted adults featured regular number-space associations regardless of hand posture (Dehaene et al., 1993; Crollen and Noël, 2015; Crollen et al., 2015). These findings suggest that younger, as opposed to older, individuals do not yet exclusively rely on an external object-centered reference frame when spatially representing numbers. They might rather depend on both internal body-centered and external frames of reference for mapping numbers onto space. The anchoring of spatial-numerical representations solely onto external coordinates might thus only gradually arise with increasing age.

Interestingly, number-space associations in 6-year-olds, but not adults, also depended on visual feedback in that no SNARC effect was observed when children were blindfolded (Nava et al., 2017). The ability to anchor numerical concepts onto an external spatial reference frame thus seems to depend on the availability of visual cues, especially at earlier developmental stages. The importance of visual experience for the development of an adult-like anchoring of numerical representations onto external space is also in line with findings in early blind adults. Namely, these individuals showed a reversed SNARC effect with crossed hands, indicating the adoption of a hand-centered reference frame during number processing (Crollen et al., 2013). Regarding these findings, children in the present study might not have been able to anchor number-space mappings onto an external reference frame when randomly generating numbers during head rotation, especially since they were blindfolded. The lack of visual feedback either completely kept them from accessing their spatial-numerical representations or induced them to rely on a rather head-centered frame of reference. This, in turn, might have masked the effect of active head rotation on RNG in the latter population. Conversely, adults probably used external spatial coordinates in that they coded numbers spatially with respect to their head facing straightforward. Left-/rightward head turns away from this position might then have induced associated spatial attention shifts on the MNL, leading to the generation of smaller/larger numbers during left/right rotation respectively. It should, however, be noted that the spatial bias in RNG in the study of Göbel et al. (2015) was evidenced despite the children having their eyes closed. This thus suggests that these younger individuals were probably able to rely on an external reference frame even in the absence of visual input. The potential reliance on a body-centered spatial reference frame during RNG due to the absence of visual feedback at earlier developmental stages is thus unlikely to account for the present null effect in the younger individuals.

An alternative explanation for the null effect in children might be that although these younger individuals could use external spatial coordinates, similarly to adults, the current instruction to generate the number while facing straightforward directed their spatial attention toward where their head was positioned at the time of number generation (i.e., straight ahead). Consequently, their left/right head turns might not have been associated with respective spatial attention shifts on the MNL. This, in turn, could then explain the absence of a significant difference in RNG between left and right rotation in the younger individuals. This explanation could also account for the spatial biases in RNG observed in the study of Göbel et al. (2015), considering that the children were positioned on their left/right and thus facing in the corresponding direction. Nonetheless, the hypothesis that spatial attention was focused straight ahead due to task instructions would anticipate a null effect also in adults, since both adults and children received the same instructions in the present investigation. It could, however, still be that the spatial attention of older as opposed to younger individuals was not restricted toward where their head was positioned at the time of number selection.

Another reason for the discrepancy between the present findings in children and those of Göbel et al. (2015) could lie in the way the effects of space were assessed. While Göbel et al. (2015) determined the impact of static left/right body orientation on RNG, we assessed the effect of active left/right head motion. In addition, it needs to be reminded that in the current set-up participants had to generate a random number during motion, while classically in the literature numbers are produced once the movement has finished (see e.g., Loetscher et al., 2008). Since participants had to generate numbers while simultaneously moving their heads left-/rightward, the current paradigm can be considered as a dual-task and was therefore probably more difficult than that implemented in previous studies. Randomly generating numbers in a situation involving lateral head turns as well as the fact that the numbers had to be produced during motion (as opposed to when the head had reached a static left/right position) might have placed additional demands on the WM system, already strained by the RNG task in itself (Jahanshahi et al., 1998; Hamdan et al., 2004). Considering that WM and executive functions have not yet fully developed in children (Luciana and Nelson, 1998; De Luca et al., 2003; Best et al., 2009), the latter participants might have been particularly negatively affected by this dual-task situation. This interpretation is supported by the greater number of omissions in children compared to adults. In addition, children featured a higher redundancy score than adults, indicating that they selected numbers less randomly. Considering that this measure is interpreted to rely on general executive functions, such as the ability to suppress response preferences created by one’s own previous output (Brugger, 1997; Baddeley, 1998; Peters et al., 2007; Terhune and Brugger, 2011), this further endorses the assumption that executive processing was particularly strained in children. Since number-space associations were previously shown to depend on available WM resources in that no SNARC effect was observed under increased WM load (Herrera et al., 2008; van Dijck et al., 2009), compromised WM resources especially in children might have prevented them from accessing spatial-numerical representations during RNG and as such precluded any spatial bias in their numerical magnitude selection during active head rotation. This could then account for the null effect in the present study, even though spatial biases were previously evidenced by Göbel et al. (2015). Overall, this interpretation further strengthens the important role of WM in the association between spatial and numerical concepts (Herrera et al., 2008; van Dijck et al., 2009, 2014; van Dijck and Fias, 2011; Ginsburg et al., 2014; Abrahamse et al., 2016; Fias and van Dijck, 2016). Considering that WM ability considerably increases between adolescence and adulthood, especially for tasks requiring retention during distraction (Fry and Hale, 2000; Gathercole et al., 2004; Ullman et al., 2014), the spatial bias in RNG during active head rotation might only arise in older children attending high-school. This would then also account for the fact that school grade did not influence the effect of active head rotation on RNG in the present group of elementary school children. In other terms, it would provide an explanation for why number selection did not significantly differ between active left/right head rotation, even in the oldest children of the current sample.

### Future Studies

To verify whether the null effect in children might be explained by their logarithmic as opposed to linear numerical magnitude representations, future studies could additionally administer a number line estimation task assessing the linearity of numerical magnitude representations. Accordingly, RNG should be least affected by active head rotation in those children featuring more logarithmic representations. The latter children should also generally produce more smaller numbers compared to their age-matched peers.

An interesting idea might also be to prime the activation of the MNL by instructing children to imagine numbers on a ruler while performing the RNG task (see Loetscher et al., 2008). This should yield valuable information regarding whether the absence of a significant effect of active head rotation on RNG in children might be explained by inefficient activation of spatial-numerical representations on the MNL during task completion.

Future studies might also envisage to replicate the present investigation without blindfolding participants. This should unravel whether the absence of visual feedback and the anchoring of numerical magnitudes onto head-centered as opposed to extra-corporal spatial coordinates in children could have accounted for the absence of a significant effect of active head rotation on RNG in the latter individuals.

Finally, to determine whether the dual-task situation and the associated compromise in available WM resources contributed to the null effect in children, one could additionally assess the children’s WM capacity. Accordingly, a null effect might only be observed in those children with weaker WM performances, while active left/right head rotation might lead to the generation of smaller/larger numbers respectively in those children with higher WM capacity, similarly to adults. Alternatively, one could assess RNG performances in a static experimental set-up not involving any left/right head motion. Considering that higher executive functions as well as WM are associated with better randomness quality (Brugger, 1997; Baddeley, 1998; Peters et al., 2007), finding evidence for better RNG performances in terms of the R score as well as the total of errors and omissions in the absence of active head rotation could then substantiate the hypothesis that WM resources were indeed likely reduced in the current dual-task paradigm, which in turn might have potentially accounted for the absence of a significant difference in RNG between left and right rotation in children.

## Conclusion

To conclude, we replicated previous findings showing an effect of active head rotation on the randomization of numbers in adults. Adults generated on average smaller numbers and the mean of FODs was smaller during left than right rotation. Importantly, by additionally studying the effects of rotation on the total as well as the absolute value of negative and positive FODs, the present study significantly advanced our understanding of how spatially directional cues such as active head rotation affect step generation and as such spatial attention shifts along the MNL. Participants produced more descending/ascending steps during left/right head rotation respectively, indicating that they shifted their attentional focus more often toward the left/right along the MNL when rotating their heads in the corresponding direction. In addition, the size of descending steps was larger (in terms of absolute value) during left than right rotation. As opposed to adults, RNG in elementary school children did not significantly differ between active left/right head rotation. Future studies should determine whether such age-related differences can be explained by developmental changes in numerical magnitude representations and/or the access to these representations or whether the null effect in children mainly resulted from the dual-task situation and the associated compromise in WM resources especially in the latter individuals.

## Author Contributions

Conceived and designed the experiments: A-MS, CtS, ClS, and MG. Analyzed the data: CG. Wrote the paper: CG, CtS, and ClS.

## Conflict of Interest Statement

The authors declare that the research was conducted in the absence of any commercial or financial relationships that could be construed as a potential conflict of interest.
